# Beyond pathogenicity: the immunomodulatory role of the type III secretion system in beneficial plant–microbe interactions

**DOI:** 10.1098/rsob.240318

**Published:** 2025-05-28

**Authors:** Iva Atanasković, Marija Nedeljković, Jelena Lozo

**Affiliations:** ^1^ University of Belgrade Faculty of Biology, Beograd, Serbia

**Keywords:** effector proteins, immunomodulation, induced systemic resistance, plant–microbe symbiosis, type III secretion system

## Abstract

The type III secretion system (T3SS) has traditionally been studied for its role in bacterial virulence. However, recent research emphasizes its dual role in beneficial interactions between bacteria and plants. This review examines the immunomodulatory functions of T3SS beyond pathogenicity and focuses on how T3SS effectors manipulate plant immune responses to promote symbioses. By comparing T3SS mechanisms in pathogenic and non-pathogenic bacteria, we aim to understand how this system enables beneficial microbes to colonize plants and improve plant growth and stress resilience. We also investigate the potential of T3SS to trigger induced systemic resistance in plants, a mechanism that could be utilized in agriculture to improve crop resistance to pathogens. The review concludes with an outlook on future research and emphasizes the need for comprehensive studies on T3SS effectors in non-pathogenic bacteria and their interactions with plant hosts.

**Figure:**
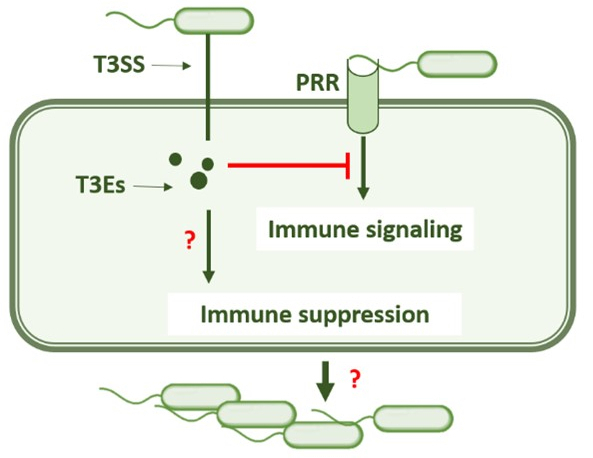

## 1. Introduction

In an evolutionary context, the distinction between microbial pathogenicity and symbiosis is often blurred, as both types of microbes use strikingly similar molecular strategies to interact with their hosts [1]. Regardless of whether a microbe enters into a beneficial or harmful relationship, it must overcome the same fundamental challenge: it must manipulate the host’s immune system to enable colonization [2]. The molecular mechanisms involved in these interactions, such as secretion systems and the effector proteins they secrete, are utilized by both pathogens and symbionts. However, while pathogens use these molecular machineries to invade and damage the host, symbiotic microbes use them to promote reciprocal relationships, leading to very different outcomes despite similar mechanisms of action. Therefore, the molecular mechanisms involved in direct host–microbe interactions need to be analysed in the context of both pathogenicity and symbiosis. This dual perspective is crucial for a more comprehensive understanding of the complex mechanisms underlying the molecular coevolution of microbes and their hosts.

Bacteria have evolved a number of secretion systems that allow them to interact with their hosts and adapt to different environments. With the identification of 11 different types of secretion systems, the complexity and diversity of these mechanisms are becoming increasingly clear, but the full extent of their functional pleiotropy has yet to be revealed [3]. Why do bacteria have so many different secretion systems, all with seemingly unique methods of transporting molecules? Do these systems fulfil highly specialized tasks, or are they capable of multifunctionality? Of the different types, three—type III, type IV and type VI—are structured like molecular syringes and allow direct contact with neighbouring prokaryotic or eukaryotic cells. The type VI secretion system (T6SS), for example, enables direct interactions between bacterial cells, often leading to competition between them [4]. In addition, all three systems can establish direct connections with eukaryotic hosts and can inject bacterial effector proteins into the host. This direct contact is crucial for influencing host–pathogen dynamics, especially in the context of bacterial virulence, where the injection of effector proteins is essential for influencing processes in the host cell [5,6]. While the role of syringe-shaped secretion systems in bacterial pathogens has been studied for decades, many questions about their function in non-pathogenic bacteria remain unanswered, even though they also occur in these microbes. Plants provide an interesting model to explore this, as bacteria in the rhizosphere can come into direct contact with root cells, suggesting that molecular syringes can inject effector proteins into the host, even in the context of a healthy plant microbiome [7]. Understanding this mechanism of interaction could provide new insights into the role of syringe-shaped bacterial secretion systems in promoting positive relationships between plants and microbes.

## 2. The dual role of the type III secretion system in pathogen virulence and plant-beneficial symbiosis

Among molecular syringes, the type III secretion system (T3SS) has been most extensively studied in the context of direct host–pathogen interactions, with numerous pathogens demonstrating its critical role in virulence [8–10]. In short, this complex nanomachine consists of more than 20 proteins that form an apparatus spanning the inner and outer membranes of Gram-negative bacteria and the membrane of the host cell. The basal body is a structural foundation of the system embedded in the bacterial membranes. The needle, which protrudes from the bacterial surface, serves as a channel for the transfer of effector proteins into the host cells. The translocation pore forms at the tip of the needle, which enables the direct injection of bacterial proteins into the host cell. These proteins, known as T3SS effector proteins (T3Es), are taken up into the secretion system by a special selection process. Effector proteins are usually kept in a partially unfolded state by chaperones that direct them to the T3SS sorting platform [11]. Once the effector is recruited, it is unfolded by the ATPase complex, passed through the needle and injected into the host. The functions of T3Es are diverse and range from influencing host immune responses to altering cytoskeletal dynamics, all of which contribute to the pathogen’s ability to invade, survive and replicate in the host [12]. The precise recruitment and hierarchical secretion of T3Es is critical for successful infection and the establishment of bacterial virulence [13].

Many examples of T3SS-reliant pathogens can be found in the genus *Pseudomonas. Pseudomonas syringae*, for example, is a plant pathogen that can infect a variety of plant hosts by injecting T3Es directly into plant cells [14]. The bacterium first colonizes the plant surface during its epiphytic phase and then invades the plant tissue to grow in the intercellular spaces, the apoplast. Once inside the plant, *P. syringae* utilizes the T3SS to release T3Es that affect the plant’s immune responses. These effectors impair the plant’s ability to recognize and respond to the presence of the bacterium, allowing the pathogen to cause infection. For example, T3Es from *P. syringae*, such as AvrPtoB, suppress plant immunity by targeting key components of immune signalling pathways, including pattern recognition receptors (PRRs) such as FLS2 [15]. Other effectors such as HopM1 and AvrE help to create an aqueous environment in the apoplast that is essential for bacterial growth [14,16]. This manipulation of the plant’s immune system creates favourable conditions for bacterial proliferation. The ability of *P. syringae* to alter both immune responses and the physical environment of the plant emphasizes the central role of the T3SS and its effectors in pathogenesis. However, T3SS is also found in non-pathogens. Genome sequencing has demonstrated the presence of complete T3SS clusters in at least 55 strains of plant-beneficial *Pseudomonas* species, but the role of the system beyond virulence remains to be elucidated [7].

**Figure 1.**
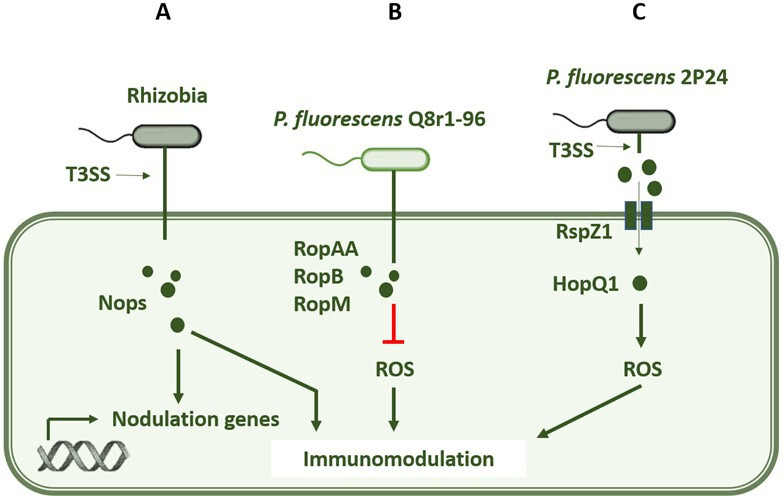
The variety of mechanisms by which T3Es can be introduced into plant cells by beneficial bacteria. (A) Rhizobia utilize the T3SS to release nodulation outer proteins (Nops), which are T3Es that drive the nodulation process in legume host plants and facilitate symbiosis. (B) *P. fluorescens* Q8r1−96 possesses a full T3SS operon, and the effectors RopAA, RopB and RopM are directly injected into plant cells. These effectors inhibit the production of reactive oxygen species (ROS), suppress plant immune responses and promote beneficial interactions. (C) *P. fluorescens* 2P24 does not have a complete T3SS translocon but utilizes the harpin protein RspZ1 to form a pore in the plant cell envelope. This allows the effector HopQ1, which is released into the apoplast, to enter the plant cells. HopQ1 stimulates ROS production and thus modulates the plant’s immune response.

The only well-documented example of a beneficial interaction involving T3SS and T3Es is found in the nodulation process of legumes (figure 1A). In this case, *Bradyrhizobium elkanii* uses T3SS effector proteins to manipulate the nodulation signalling pathway of its plant host [17]. Rather than relying solely on rhizobial Nod factors (NFs) to initiate nodulation, *B. elkanii* can bypass this traditional signalling mechanism by using its T3SS to promote nodulation directly. The effector proteins involved in this process are known as nodulation outer proteins (Nops). Studies have shown that *B. elkanii* can form nitrogen-fixing nodules even in the absence of functional NFs or their corresponding plant receptors, as demonstrated in *Glycine max nfr* mutants. Nops of *B. elkanii* can activate nodulation genes such as ENOD40 and NIN, which are important for nodule organogenesis. Nops can also modulate the plant’s immune system to create a favourable environment for symbiosis by reducing plant defence responses and facilitating bacterial penetration into the plant’s root system [17]. This dual function of T3SS, both in promoting nodulation and modulating plant immunity, highlights the broader potential of T3SS beyond pathogenicity.

Based on what is known about the function of T3SS in pathogens and in the nodulation process, several hypotheses can be formulated about the function of this system in symbiotic interactions in non-nodulating microbes, such as the plant-beneficial *Pseudomonas* species mentioned above. The first question that arises is: what is the final destination of T3Es in these non-pathogenic bacteria? The T3SS can target any eukaryotic cell, including protist or fungal cells, and could serve as a defence mechanism against predation by protists [18]. This system could also facilitate beneficial interactions between plants and fungi, such as promoting mycorrhization [19], or it could protect the host plant by killing pathogenic fungi [20]. Indeed, studies have shown that some T3SS-bearing *Pseudomonas* strains are enriched in the mycorrhizosphere, suggesting that T3SS could contribute to supporting mycorrhizal symbioses by interacting with the plant or fungal cells involved [21]. However, these scenarios do not fully explain the responsiveness of T3SS in beneficial bacteria to plant signalling [22,23]. In addition, there is some biochemical evidence that T3Es are transferred from beneficial *Pseudomonas* into plant cells [24], which has not yet been demonstrated in the case of protists and fungi. Therefore, it seems plausible that, at least for some non-pathogen *Pseudomonas* isolates, the target of T3Es is the plant host itself.

While there is some biochemical evidence that T3Es are transferred from beneficial *Pseudomonas* into plant cells, direct evidence remains limited, and more data on T3SS functionality in beneficial bacteria are needed to support this idea. One example is *P. fluorescens* Q8r1−96, a beneficial rhizobacterium that possesses a complete T3SS operon similar to the system found in the pathogen *P. syringae* or in rhizobia (figure 1B). Effectors from *P. fluorescens* Q8r1−96, RopAA, RopB and RopM, were fused to adenylate cyclase at their C termini and infiltrated into *Nicotiana tabacum* cv. *Xanthi* leaves. Leaf discs were harvested and cyclic AMP levels were measured, demonstrating that the effectors were successfully transported into the plant cells. Effectors were not only transported but were also functional, as they were shown to inhibit the production of reactive oxygen species (ROS) and thus modulate the plant’s immune response [24]. Another example, albeit with a different mechanism of effector transport, comes from *P. fluorescens* 2P24 (figure 1C). In contrast to Q8r1−96, this strain lacks a complete T3SS operon, in particular the proteins of the effector translocation pore that form the needle tip. However, it encodes a harpin protein, RspZ1, which can also form a pore in the plant cell envelope [25]. In this case, the secretion mechanism is that the effector HopQ1 is released into the apoplast of the plant. From there, it enters the plant cells through the pore formed by the harpin. Remarkably, HopQ1 not only entered the plant cells but also had an immunomodulatory effect by stimulating ROS production, which is an essential component of the plant defence response [25]. These examples emphasize the diversity of mechanisms by which T3Es can be introduced into plant cells by beneficial bacteria and highlight the complex and multifaceted role that these effectors can play in plant–microbe interactions. In all three examples, the T3SS is involved in modulating the plant’s immune response. This raises new questions: what are the immunomodulatory effects of T3Es in non-pathogens, and what are the ultimate purpose and outcome of these effects in the context of symbiosis?

## 3. Decoding the immunomodulatory role of the type III secretion system in beneficial bacteria

### 3.1. Beneficial immunosuppression: how bacterial type III secretion systems enhance plant–bacterial symbiosis

Beneficial microbes that form the plant’s microbiome can either evade or suppress the plant’s immune system to successfully colonize the host [26]. This evasion of the immune system is crucial for the formation of beneficial interactions without triggering a full immune response that could hinder the colonization process. To achieve this, microorganisms use various strategies, such as the diversification of microbe-associated molecular patterns (MAMPs), the microbial molecules that can be recognized by the host’s PRR receptors to trigger immune responses [27]. One example of the evasion of the immune system is *Sinorhizobium meliloti*, a nitrogen-fixing symbiont of legumes. The flagellin of *S. meliloti* has evolved to evade recognition by the plant’s PRRs, such as FLS2, thus avoiding immune activation in the host plant *Arabidopsis thaliana* [28]. Similarly, *Mesorhizobium loti*, another plant bacterium, can avoid recognition by the immune system in its host *Lotus japonicus* due to divergences in its flagellin sequence [29]. However, it is not always possible to evade the immune system, as plants are constantly diversifying their PRRs in an evolutionary arms race with microbes. This diversification enables plants to recognize even previously evasive beneficial microbes. Thus, despite their efforts to avoid detection, some beneficial microbes can still be recognized due to the plant’s evolving immune system. As a result, many beneficial microbes have developed additional strategies to actively suppress the plant’s immune response. These strategies are based on the inhibition of various signalling molecules that are activated during MAMP recognition, e.g. by suppressing ROS bursts, targeting kinases in immune signalling cascades and modulating hormonal immune signalling. To this end, microbes produce effector proteins and organic molecules that are secreted either into the apoplast or directly into the cytoplasm of the host cells [26].

**Figure 2.**
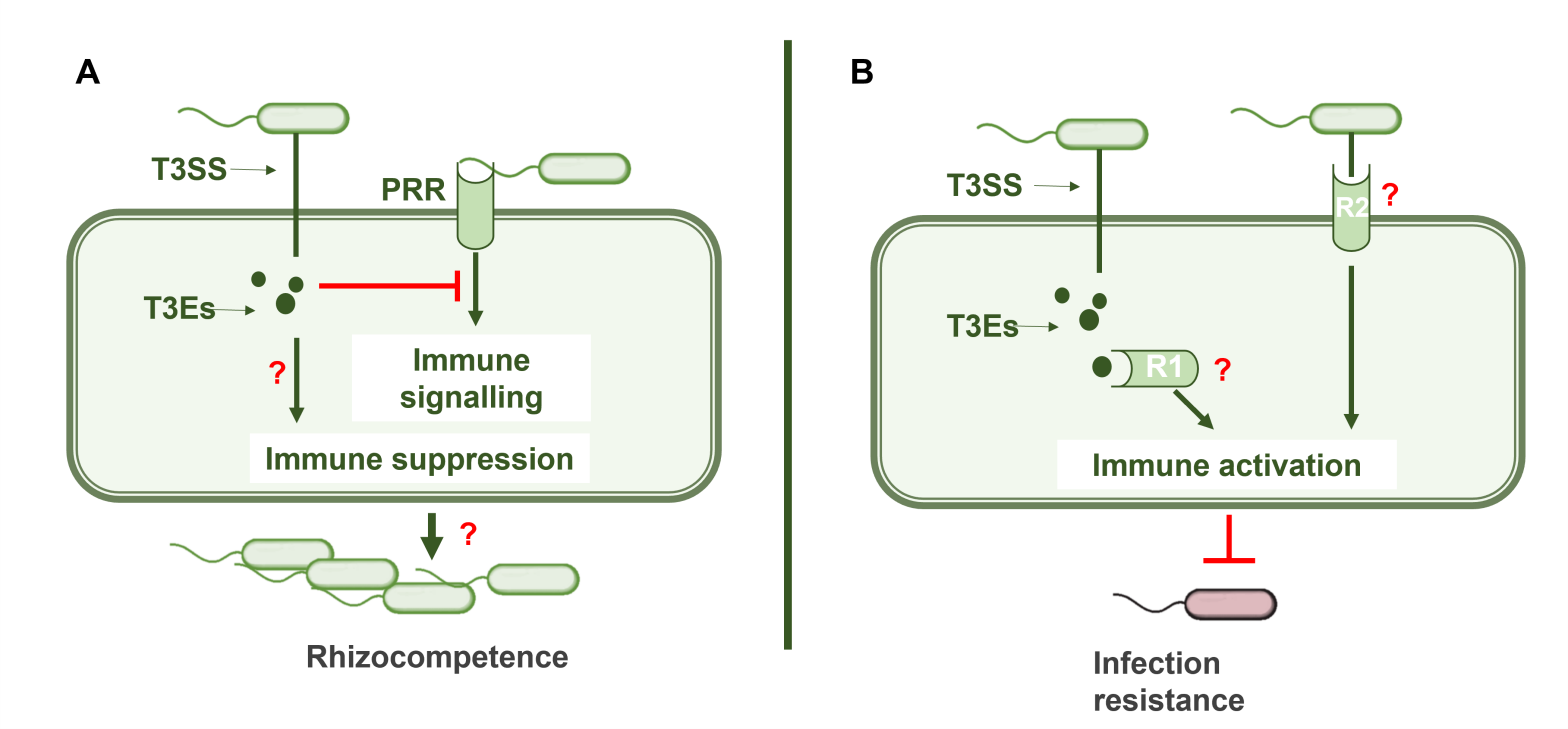
Models of T3SS-mediated immunomodulation by beneficial bacteria. (A) Suppression model: beneficial bacteria use T3Es to suppress the plant immune response by inhibiting PRR activation. This suppression of the immune system may allow the bacteria to evade detection and increase their rhizocompetence, promoting successful colonization of the rhizosphere. (B) Activation model: beneficial bacteria can also activate plant immunity by two possible mechanisms. First, T3Es can be recognized by intracellular receptors (R) such as the NBS-LRR proteins and thus trigger ETI. Secondly, structural components of the T3SS itself can be recognized by extracellular PRRs (R), which activate MAMP-triggered immunity. Although the molecular nature of these receptors (R1 and R2) is not yet fully understood, immune activation by beneficial bacteria can ultimately enhance host plant resistance to pathogens.

In beneficial bacteria, T3SS and its effectors play a central role in modulating hormonal signalling pathways and suppressing the immune response [7,17]. Metagenomes of root microbiomes of various plant species such as cucumber, wheat, citrus and barley show significant enrichment of T3SS genes at the community level, and these genes may be involved in the suppression of root immune responses [26]. Beneficial bacteria can exploit the immunomodulatory activity of T3Es to promote host plant colonization by manipulating hormone signalling cascades and blocking immune defence mechanisms [7]. However, little is known about the mechanisms of beneficial immunosuppression. In nodulating rhizobia, Nop effectors play a direct role in the suppression of PRR-mediated immune signalling [30]. NopM, an effector secreted by *Sinorhizobium* sp. NGR234, functions as an E3 ubiquitin ligase and is critical for normal nodulation in *Lablab purpureus* [31]. In *Nicotiana benthamiana*, NopM was shown to suppress flg22-induced ROS bursts [14]. ROS are produced within minutes of PRR-mediated MAMP recognition and function as important signalling molecules in plant immunity, implying that the immunosuppressive activity of NopM may be important for evading the immune system in the nodulation process. Some examples of this phenomenon have also been described in plant-beneficial *Pseudomonas* strains, but details on the mechanism are still lacking. *P. fluorescens* Q8r1−96, which secretes RopAA, RopB and RopM (figure 1B), needs these effectors to suppress plant immune responses such as the hypersensitive response and oxidative burst, key components of MAMP-induced immunity [24]. Another example is *P. simiae* WCS417, in which the T3SS plays a role in the partial suppression of MAMP-induced immunity, as shown by transcriptomic studies in *A. thaliana* [32]. In summary, beneficial bacteria, like pathogens, can use T3Es to block PRR-mediated immune signalling (figure 2A), but the mechanistic details of this process remain unclear.

Another critical question is the biological role of T3SS-mediated suppression of plant immunity. In beneficial microbes, immunosuppression is thought to promote rhizocompetence, or the ability of bacteria to colonize the rhizosphere. However, it is not yet known whether T3SS functionality is essential for rhizocompetence. This has so far only been investigated in one study, in which deletion of the T3SS in *P. fluorescens* Q8r1−96 had no effect on the ability of the bacterium to colonize plant roots [24]. In this study, long-term rhizosphere colonization experiments were conducted with wheat and pea in raw soil. The T3SS mutant of Q8r1−96 was tested both in solo colonization experiments and in direct competition with the wild-type parent strain. Bacterial populations were determined by PCR-based endpoint dilution assays, which revealed no significant differences in colonization efficiency between the mutant and wild-type strains. However, further studies are required to fully understand the role of T3SS in rhizocompetence across a broader range of beneficial bacterial species and colonization conditions. For example, it could be investigated whether T3SS-mediated immunosuppression affects the colonization ability of neighbouring microorganisms in the rhizosphere, potentially creating a more favourable environment for different beneficial bacteria. Comprehensive studies are needed to evaluate the colonization efficiency of T3SS deletion mutants in different bacterial species and how T3SS mutations affect the entire microbiome. In addition, detailed research into the specific T3Es involved in these processes and their effects on plant immune signalling will help to elucidate the full range of T3SS functions in beneficial plant–microbe interactions.

### 3.2. The immunostimulatory role of the type III secretion system in enhancing plant resistance to infection

Beneficial microbes can initially be recognized by plants as potential invaders, which triggers an immune response [33]. However, at later stages of the interaction, the mutualists are able to evade or avoid the plant’s defence responses to allow successful colonization. Therefore, some beneficial microbes can stimulate the host’s immune response for short or longer periods of time, and this effect can also promote colonization by the microbe [34]. For example, *Bradyrhizobium japonicum*, a nodulating bacterium, induces strong expression of defence‐related genes in the early phase of nodule formation in soya bean root hair cells [35]. Examples can also be found in the genus *Pseudomonas*: the cellular components of two T3SS-positive plant growth-promoting isolates, *P. simiae* WCS417 and *P. capeferrum* WCS358, trigger immune responses in *A. thaliana* roots and tobacco cells, including ROS production, MAMP‐dependent gene expression and callose deposition [36]. Overall, these studies show that root immune responses are indeed triggered by beneficial microbes. However, this activation appears to be largely restricted to the early stages of beneficial interactions, suggesting that beneficial microbes may actively suppress root immunity as the association progresses [26].

There is some evidence that the T3SS in beneficial bacteria can be required for immune system activation, e.g. ROS bursts and local hyperactivation of the immune system known as hypersensitive response [23,25]. The mechanism behind this remains an open question. One possibility is that multiple T3SS-related antigens are recognized by the plant’s immune system (figure 2B). Similar to the T3Es of the pathogen *P. syringae*, the T3Es of beneficial bacteria could be recognized by intracellular receptors in the plant, such as proteins containing a nucleotide binding site (NBS) and a leucine-rich repeat (LRR) domain, which trigger effector-triggered immunity in the host [37]. This mechanism would suggest that plants have the ability to recognize bacterial effectors, even from mutualistic bacteria, to trigger a controlled immune response. Another hypothesis is that the structural components of the T3SS present on the bacterial cell surface could be recognized extracellularly by PRRs. These PRRs typically recognize conserved microbial patterns, and their ability to recognize T3SS structural elements in beneficial bacteria is still unexplored. In evolutionary terms, T3SS is closely related to the bacterial flagellum, and the structures of these two molecular machines are highly conserved [38]. Flagellin is a known MAMP that can be recognized by PRRs in plants and animals [39]. Therefore, it is plausible that the needle of the T3SS or other conserved components could similarly act as MAMPs and trigger immune responses through PRR recognition.

### 3.3. Exploring the type III secretion system as a potential inducer of systemic resistance against pathogen infections

**Figure 3.**
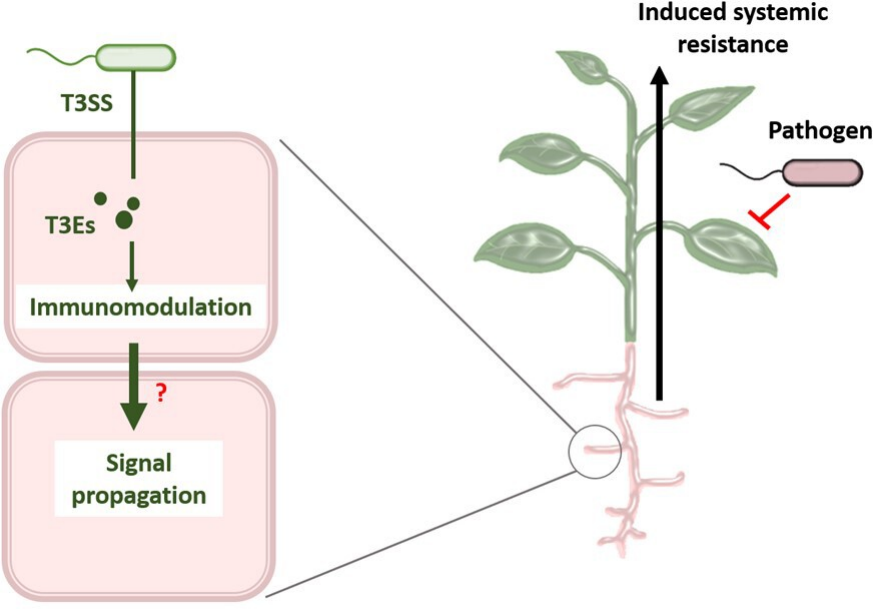
Hypothetical mechanism of ISR mediated by T3SS in beneficial bacteria. Beneficial bacteria can utilize T3SS to inject T3Es into root cells to modulate the immune response of plants. This immune modulation could generate systemic signals such as plant hormones that spread throughout the plant and increase resistance to subsequent pathogenic infections in above-ground tissues. Further research is needed to confirm this hypothesis and to understand the potential systemic effects of T3SS-mediated immunomodulation.

Regardless of the mechanism, immune modulation triggered by beneficial bacteria could serve an important purpose (figure 3). Beneficial bacteria could help to activate the plant’s immune system, leading to increased resistance to pathogenic infections. This phenomenon, known as induced systemic resistance (ISR), allows the host plant to better respond to subsequent attacks by pathogens while maintaining the beneficial association with the microbe [40]. Therefore, understanding the dual role of T3SS in both immunosuppression and immune activation is crucial to fully understand how beneficial bacteria colonize their hosts and contribute to plant health. Various bacterial molecules can act as ISR triggers. For example, flagellin, a previously mentioned MAMP, can trigger ISR when recognized by PRRs. Other triggers include lipopolysaccharides, cyclic lipopeptides and quorum sensing molecules [39]. These molecules, produced by beneficial bacteria, can trigger defence responses in plants, often via pathways involving jasmonic acid and ethylene, which are critical for ISR [40]. Since T3SS is evolutionarily related to the flagellum [38] and is structurally conserved between pathogenic and non-pathogenic bacteria [41], it also has the potential to act as an immune trigger. However, little is known about the role of T3SS in non-pathogenic bacteria as an ISR inducer.

In the case of the pathogen *P. syringae*, T3SS effectors can suppress plant immunity. In non-compatible hosts in which the T3SS effectors cannot suppress immune responses, the T3SS itself can act as an antigen and trigger an immune response, thereby promoting resistance to infection [42]. In particular, components of the T3SS can trigger ROS production, an important marker for the activation of ISR. This was observed in *Nicotiana benthamiana*, where *P. fluorescens* expressing the T3SS of *P. syringae* elicited a strong response to ROS production [43]. Stimulation of the immune system by T3SS has also been described in animal pathogens. The introduction of the needle tip translocon components into the host plasma membrane can lead to activation of the inflammasome. Specifically, the NLRC4 inflammasome is activated in response to T3SS-dependent pore formation, leading to the activation of caspase-1 and the production of pro-inflammatory cytokines such as IL-1β. This has been observed in pathogens such as *Salmonella*, *Shigella* and *Pseudomonas aeruginosa* [44]. However, the role of T3SS as an antigen that triggers ISR in plants has not been thoroughly investigated in plant-beneficial root colonizers. For example, it is not known what effects T3SS-dependent pore formation has in plant hosts, whether T3SS from beneficial bacteria can stimulate ISR or which specific T3Es from beneficial bacteria can have this effect. Further research in this area could provide valuable insights to improve plant immunity in agriculture.

## 4. Future directions in unravelling the functional dynamics of the type III secretion system

Many questions about the functional dynamics of the T3SS in plant-beneficial bacteria, especially in non-nodulating genera such as *Pseudomonas*, remain unanswered. While it is known that these bacteria possess T3SS and that the system is expressed on the plant surface [22], crucial biochemical evidence confirming the injection of effectors into host plant cells is limited. In addition, a comprehensive functional analysis with T3SS or T3E deletion mutants and their effects on the host plant phenotype still need to be thoroughly explored.

There is evidence that the T3SS plays an immunomodulatory role in beneficial bacteria, similar to plant pathogens. In pathogens, this immunosuppressive effect helps to establish the infection. In beneficial bacteria, on the other hand, immunomodulation can either stimulate or suppress the host’s immune responses, thus facilitating the establishment of a symbiotic relationship. This dual functionality raises many questions. How can a single system both activate and suppress the immune response? Is this dual action universal, or is the immune response stimulated or inhibited depending on the bacterial species and host plant? Furthermore, what is the relationship between immune modulation and successful colonization of the host? For example, if a beneficial *Pseudomonas* strain colonizes the plant root and stimulates systemic immune responses through T3SS, how does it still manage to successfully colonize the plant and evade host immunity? This raises further questions about possible local effects at the root that could support colonization despite systemic immune activation and whether T3SS plays a role in these processes. To comprehensively address these questions, future studies need to take a systematic approach. T3SS deletion mutants of different plant-beneficial species isolated from the microbiome of healthy plants are needed. Research should focus on investigating the effects of T3SS on host colonization efficiency and immune response at both local (root) and systemic (above-ground tissue) levels. Furthermore, these effects need to be studied in a time-dependent manner, from the first stages of colonization to the point where a stable symbiotic relationship is established. In this way, we can better understand the temporal and spatial dynamics of T3SS-mediated immune modulation and how this system balances immune activation and suppression to promote a beneficial association between bacteria and host plants.

## Data Availability

This article has no additional data.
